# Words affect visual perception by activating object shape representations

**DOI:** 10.1038/s41598-018-32483-2

**Published:** 2018-09-20

**Authors:** Samuel Noorman, David A. Neville, Irina Simanova

**Affiliations:** Radboud University Nijmegen, Donders Institute for Brain, Cognition and Behaviour, Centre for Cognition, Nijmegen, The Netherlands

## Abstract

Linguistic labels are known to facilitate object recognition, yet the mechanism of this facilitation is not well understood. Previous psychophysical studies have suggested that words guide visual perception by activating information about visual object shape. Here we aimed to test this hypothesis at the neural level, and to tease apart the visual and semantic contribution of words to visual object recognition. We created a set of object pictures from two semantic categories with varying shapes, and obtained subjective ratings of their shape and category similarity. We then conducted a word-picture matching experiment, while recording participants’ EEG, and tested if the shape or the category similarity between the word’s referent and target picture explained the spatiotemporal pattern of the picture-evoked responses. The results show that hearing a word activates representations of its referent’s shape, which interacts with the visual processing of a subsequent picture within 100 ms from its onset. Furthermore, non-visual categorical information, carried by the word, affects the visual processing at later stages. These findings advance our understanding of the interaction between language and visual perception and provide insights into how the meanings of words are represented in the brain.

## Introduction

Humans possess the unique ability to label objects. How does this ability transform cognition and perception? This question goes to the core of what it means to be human. Among philosophers^1–5^ and cognitive scientists^6–9^, many have commented on the unique way in which verbal labels enable humans to access and manipulate mental representations. However, only recently the interplay between verbal labels, concepts, and percepts at the neural level has become a subject of research^10^. An important empirical question is: what kind of representations are activated by linguistic labels? Here we address this question by studying how labels affect the processing of upcoming visual information. Namely, we test the hypothesis that words guide visual perception by activating information about visual object shape.

Several studies have shown that labels facilitate object recognition^6,11–17^ and visual object detection^18–21^. It has been proposed that cueing an object presentation with a word leads to more efficient visual processing compared to cueing with other types of cues^12,14^. Consider a classical experiment, in which one hears an auditory cue and then sees a picture. The cue can be either an object label or an equally familiar and unambiguous nonverbal sound. The task is to respond “yes” if the cue and the picture match (e.g., a picture of a dog follows a barking sound), and “no” otherwise. Using this task, Lupyan and Thompson Schill^13^ found that linguistic cues lead to faster and more accurate responses compared to non-linguistic sounds.

A more recent study by Boutonnet and Lupyan^22^ investigated the neural correlates of this label advantage effect. Participants performed the same cue-picture matching task, while their electroencephalography (EEG) signal was recorded. Analysis of the event-related potentials (ERPs) in response to the target pictures revealed the label advantage as early as 100 ms after picture presentation, in the time window of the P1 evoked component. In particular, pictures that were cued by labels elicited an earlier and more positive P1, compared with the same pictures cued by nonverbal sounds. Further, the word-picture congruency was predicted from the P1 latency on a trial-by-trial basis, but only in the label-cued trials. These results indicate that verbal cues provide top-down guidance on visual perception, and change how subsequently incoming visual information is processed early on. This suggestion is in line with the recent advance in visual perception research, which shows that object recognition is afforded by bidirectional information flow^23–25^.

Boutonnet and Lupyan^22^ conclude that labels generate categorical predictions. However, they do not dissociate between the effects of low-level, purely visual and higher-level semantic information. The distinction is relevant for understanding what type of representation is activated by a verbal label. Previous studies have addressed this question by tracking patterns of eye fixations on objects in response to spoken words. When presented with an array of objects following a target word people typically fixate on objects that are visually related to the target. For example, when hearing the word “belt”, they would fixate on a visually similar picture of a snake. However, people also show a substantial bias in orienting toward semantically related objects, e.g. a picture of socks after hearing the target word “belt”. These observations have led to the cascaded model of visual-linguistic interactions^26–29^, which suggests that words evoke both visual and semantic representations. However, the dynamics of these activations remain poorly understood. Huettig and McQueen^27^ showed that activation of semantic and visual representation occurs largely simultaneously (see also Ferreira *et al*.^26^). More recently De Groot *et al*.^30^ found that the bias in orienting towards semantically related objects occurs later than biases towards visually similar objects. Moreover, the temporal dynamics of the semantic bias stayed the same regardless of the presence of visual competitors, suggesting that the semantic information is accessed independently of the visual bias. Eye-tracking, however, can only provide an indirect measure of the temporal dynamics of cognitive processes, and does not reveal the underlying neurocognitive mechanisms. In the present study, we use the advantage of EEG to address this problem.

We specifically dissociate category information from visual object shape. Category distinctions are typically highly correlated with object shape^31^. When children learn to name objects, they pick shape as the most relevant feature: children are most likely to extend a new word to a new object that is similar to the word’s original referent in shape, rather than in colour, texture, etc. (see e.g.^32,33^). The rapid growth of infants’ vocabulary at the age of 18–24 months is strongly correlated with the ability to categorise objects based on shape^32–39^. A recent study with 3-year old children addressed the interaction between language and visual shape perception using a visual search paradigm^40^. Children first saw a cue picture of an object, and then had to identify this object among an array of distractors, with either similar or dissimilar shapes. On half of the trials the cue picture was accompanied by the object’s name. The reaction times showed the label advantage: children were faster in identifying the target when first hearing its name, compared to the no-name trials. They were also faster in identifying the target among the distractors with dissimilar shapes. Most notably, there was an interaction between shape and language: labels especially enhanced the target detection among objects with dissimilar shape. This indicates that words might guide visual search towards detection of object shape.

In the present study, we aim to disentangle the visual and semantic category contributions in the effect of words on object recognition at the neural level. Based on the study by Boutonnet and Lupyan^22^ and the evidence from the literature on language development outlined above^32–40^ we hypothesise that verbal cues activate representations of their referents’ visual object shape, which affects early stages of the upcoming stimulus’ visual processing. Further, based on the evidence from the eye-tracking study by de Groot *et al*.^30^, we hypothesise that the effects of visual shape are separable from those of category, both in timing and topography of EEG.

To test these hypotheses, we created a set of object pictures from two categories with varying shapes, while carefully controlling other visual variables. We obtained subjective measures of the shape similarity and category similarity between the objects, based on participants’ ratings. We then conducted a word-picture matching experiment, while recording participants’ EEG, and assessed how the shape and semantic category information carried by the cue word affects the processing of the upcoming picture’s shape and semantic category, respectively. Following our hypotheses, we predicted that the behavioural measures (response times) would be explained by both shape and category similarity between the cues and the pictures. To address the shape or the category similarity effects at the neural level, we employed a novel similarity-based analysis combined with non-parametric cluster-based statistics. This approach allows us to evaluate the effects of shape and category similarity without an explicit assumption on their timing or topography. We expect that early event-related responses would be explained by the shape similarity between the cues and the pictures. Moreover, we expect to see later, shape-independent effects of the category similarity. If our hypothesis does not hold, we expect the effects of visual and category similarity to occur simultaneously and have similar topography^26,27^.

## Methods

### Participants

20 native Dutch speakers (11 males, aged 23 ± 2.4), recruited via the Radboud Research Participation System, participated in the study. All participants were right-handed, and reported that they did not suffer from any psychological or neurological disorders. The experiments were approved by the local ethics committee (Commissie Mensgebonden Onderzoek Regio Arnhem-Nijmegen), and all the subjects gave written informed consent prior to the experiment. All experiments were performed in accordance with relevant guidelines and regulations. Subjects received either monetary compensation or course credits for their participation.

### Stimuli

**Figure 1:**
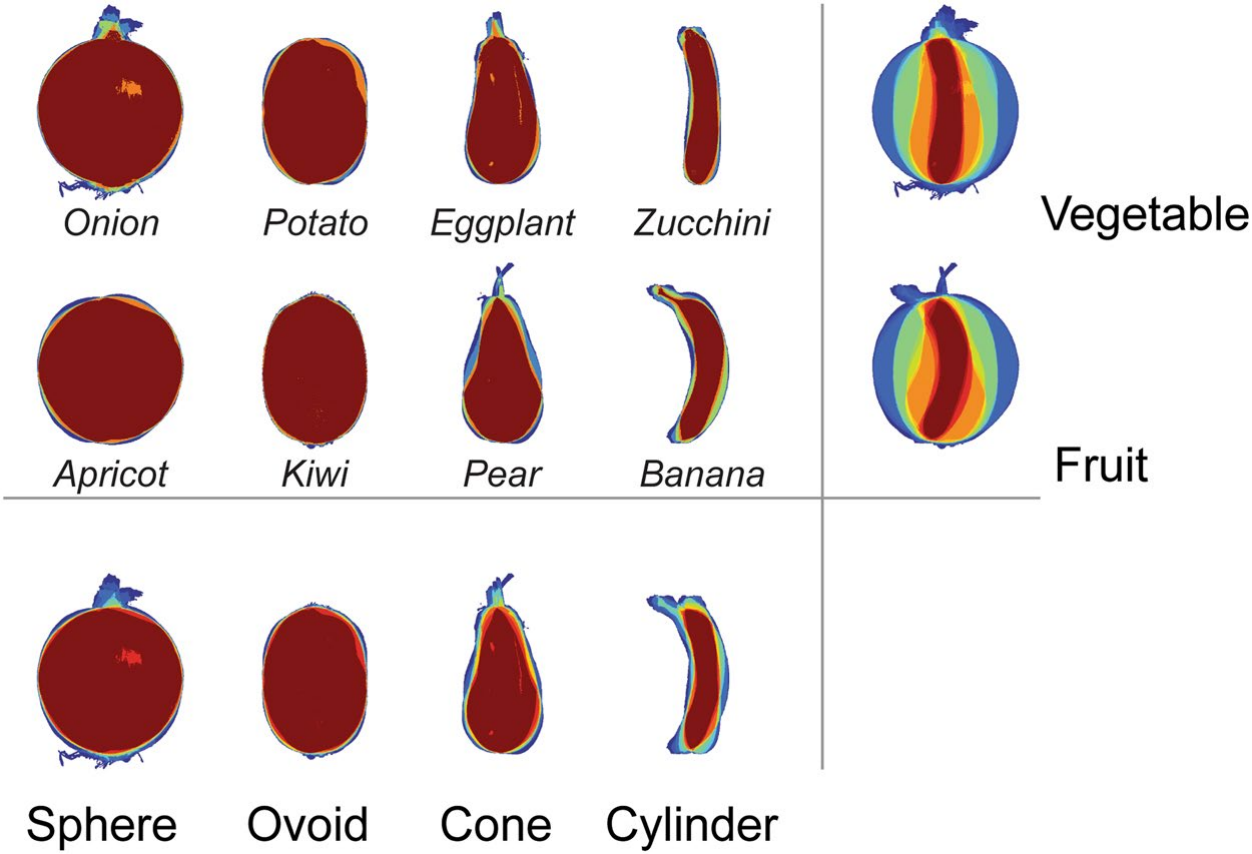
The pixelwise overlap computed by summing all stimuli images per object, per category (the rightmost column), and per shape type (the bottom row). There were eight different objects and 40 unique object images. The objects comprised of two categories and four shape types.

Four different fruits (apricot, kiwi, pear, banana) and four different vegetables (onion, potato, eggplant and zucchini) were selected as target objects. Within the category, each object had unique shape property, thus comprising four pairs of objects with similar shape: sphere, ovoid, cylinder and cone. We used detailed photographs of these fruits and vegetables, obtained in Google search with the option “free for use, share and modification”. There were five images per object, thus 40 different images in total. The images were greyscaled, placed on the white background to fit the frame of 333 × 333 pixels (7° of visual angle). Luminance and spatial frequency were matched using the Shine toolbox^41^. Figure 1 illustrates the shape overlap of the images. Additionally, for each object an auditory name (spoken Dutch words) was recorded digitally at 16 bits with a sampling rate of 44.100 Hz). The mean auditory word length was 698.7 ms ± 150.8 ms.

### Behavioural similarity rating

**Figure 2:**
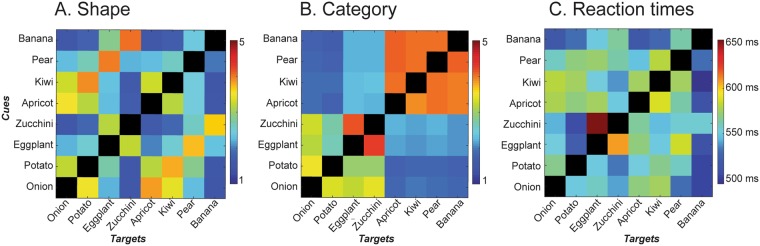
Similarity matrices and reaction times. Prior to the experiment, participants completed the rating task where they indicated how similar each target object is to each cue word, on a scale from one to five, separately for the shape and for the category dimensions. Panels A and B show the similarity ratings averaged per cue-target pair and across subjects, for the Shape (**A**) and Category (**B**) dimensions (red represents large similarities). During the main experiment, participants replied with the button press if the target object matched or mismatched the cue word. Panel C shows the reaction times (in ms) averaged per cue-target pair and across subjects (red represents slower reaction times). Note that in all reported analyses we used individual, rather than group-averaged similarity ratings and reaction times. The group-averaged data are only shown here for illustration. The similarity data (**A**,**B**) and the reaction times (**C**) on the diagonals of the matrices, which correspond to the congruent pairs, are not shown, because only incongruent trials were used for the analysis.

The object similarity judgements for both the category and shape dimensions were collected from all participants. Participants completed the rating task prior to the EEG task. During the rating task they were presented with a word and an array of objects, and were asked to indicate how similar each object is to the word, on a scale from 1 to 5. Each participant rated all 40 images relative to all eight words. The rating procedure was repeated twice, once for the shape dimension and once for the category dimension (Fig. 2).

### EEG experiment

Participants completed 960 trials of the word-picture matching task. On each trial, participants heard a cue word (a fruit or vegetable name), followed by a picture after one second delay. They were instructed to respond via button press whether the picture matched the word (yes or no). The picture remained visible for 1000 ms. In 30% of trials (congruent trials) the picture matched the cue. In the remaining 70% of trials (incongruent trials), the picture was of another fruit or vegetable. Each incongruent combination of a cue word and a target picture was repeated 12 times, and each congruent pair 36 times. The order of trials was randomised across participants. The total experiment duration was approximately one hour, and participants took nine short breaks of 30 seconds.

### EEG recording and processing

Continuous EEG was registered using a 64 channel ActiCap system (Brain Products GmbH) filtered at 0.2–200 Hz and sampled at 500 Hz with the BrainVision Recorder Professional software (Brain Products GmbH). An equidistant electrode cap was used to position 60 electrodes on the scalp. EEG data were recorded against the reference at the right mastoid; an additional electrode measured the voltage on the left mastoid, and the data were offline converted to a linked-mastoids reference. Bipolar electrooculogram (EOG) was computed using electrodes that were placed horizontally and vertically around the eyes. For all subsequent processing and analysis, we selected only incongruent trials in which participants correctly identified word-picture mismatch within 1500 ms after the stimulus onset (98 ± 1.6% of all incongruent trials per subject, on average). Data segments of 1200 ms, starting from 200 ms before image onset, were extracted. Segments containing eye-movements, or muscle artifacts were identified based on signal variance. Identified segments were inspected visually and rejected if contamination with artifacts was confirmed. On average, 8.27% of the trials were rejected. In the remaining data, line noise (50 Hz and harmonics) was removed using a discrete Fourier transform filter. The data were subsequently bandpass filtered from 0.5 to 40 Hz and baseline corrected to the 200 ms before image onset. Finally, using independent component analysis, artifacts caused by blinks and other events not related to brain activity were removed from the data. All offline data processing was performed using MATLAB R2015A and FieldTrip^42^.

### Data analysis

#### Step 1: Computing correlations

**a)** **Reaction time data:** For each participant, the mean reaction times were computed for each type of incongruent word-picture combination. This resulted in a vector of 56 mean RT values per participant. We then used a correlation analysis to test if the RTs are explained by the similarity between cues and pictures. To elaborate, for each participant, we computed a Spearman rank correlation between the word-picture similarity and the corresponding RTs. We tested two different similarity models: i) subjective shape similarity per subject and ii) and subjective category similarity per subject.

**b)** **EEG data**: For each participant, ERPs in response to the picture presentation were computed for each type of incongruent word-picture combination. Thus, the averaging resulted in 56 ERP waveforms for each channel. We further used a correlation-based analysis to test if the pattern of the evoked responses across the word-picture pairs could be explained by the similarity between cues and pictures. We tested two similarity models: i) subjective shape similarity per subject and ii) subjective category similarity per subject. We thus applied exactly the same procedure as described above in the RT analysis, but now linear Spearman rank correlations were calculated between the similarity ratings and the ERP’s. Correlations were computed for each channel and time point. This resulted in a channel x time matrix of correlation coefficients for each participant and for each model. A similar analysis approach has been used in a priming experiment before^43^.

#### Step 2: Inferential statistics

**a)** **Reaction time data:** To statistically quantify the correlation effects at the group level, we performed a one-sample t-test against the hypothesis that the mean group correlation is 0. We performed this test separately for each similarity model.

**b)** **EEG data:** The step of computing correlations between the ERPs and similarity ratings, described above, resulted in a channel x time matrix of correlation coefficients for each participant. At the group level, we aim to test if the correlations in each channel x time sample are different from zero. However, testing each time x channel sample independently leads to massive multiple comparisons. To account for the multiple comparisons problem, we used nonparametric cluster-based permutation statistics approach^44^. In this method, the complete channel x time matrix is tested by computing a single test statistic, and therefore, the multiple comparisons problem is resolved. We elaborate on this procedure in the following paragraphs.

We followed the procedure described previously^45,46^. We first computed a paired-sample t-test for each channel x time point, where we compared the correlation coefficients from 20 participants with a vector of 20 zeros. All *t* values above a threshold corresponding to an uncorrected *p* value of 0.05 were formed into clusters by grouping together adjacent significant channels (based on a minimum neighbourhood distance between the electrode sites) and time samples. This step was performed separately for samples with positive and negative *t* values (two-tailed test). The *t* values within each cluster were then summed to produce a cluster-level *t* score (cluster statistic).

This statistic was entered in the cluster-based permutation procedure^44^. To obtain a randomization distribution to compare with the observed clusters, we randomly exchanged the condition labels between the true and null conditions (that is, the vector of zero correlations, same as described above). We then computed the paired sample t-test. This procedure is equivalent to randomly multiplying the correlation values by 1 and −1, and computing a one-sample t-test against zero^45,46^. This step was repeated across 5000 permutations of the data. For each permutation, we computed the cluster-sums of subthreshold t-values. The most extreme cluster-level *t* score on each iteration were retained to build a null hypothesis distribution. The position of the original real cluster-level *t* scores within this null hypothesis distribution indicates how probable such an observation would be if the null hypothesis was true (no systematic difference from 0 in correlations across participants). Hence, if a given negative/positive cluster had a cluster-level *t* score lower/higher than 97.5% of the respective null distribution *t* scores, then this was considered a significant effect (5% α level).

For the final analysis, we focused on the time interval from 0 to 600 ms after the target stimulus onset. The sensitivity of the cluster-based ERP statistics depends on the length of the time interval that is analysed. To increase the sensitivity of the statistical test, it is therefore recommended to limit the time interval on the basis of prior information about the time course of the effect. Since we were particularly interested in the early effects, we chose to run separate analyses in the early (0–300 ms) and late (300–600 ms) intervals after the stimulus onset.

## Results

### Behavioural results

The average shape similarity rating across all cue-target pairs across all subjects was 2.55 ± 0.51; its average range was 2.90 ± 0.67 (Fig. 2, Panel A). The average category similarity rating across all cue-target pairs across all subjects was 2.72 ± 0.51; and its average range was 2.80 ± 0.93 (Fig. 2, Panel B). The average reaction time (RT) across all trials across all subjects was 554 ± 107 ms; the average range of RT across all trials and all subjects was 241 ± 73 ms (Fig. 2, Panel C). We found a robust correlation between reaction times and subjective shape similarity (mean correlation between individual subjective shape similarity and reaction times M = 0.36 ± 0.22, significantly different from zero across subjects t(19) = 6.95, p < 0.001, d = 1.55). The more similar the target object was to the cue word’s referent in terms of shape, the longer it took for participants to identify incongruence. At the same time, the reaction times were not correlated with the category similarity ratings (M = 0.007 ± 0.16, t(19) = 0.18, n.s.).

### EEG results

**Figure 3:**
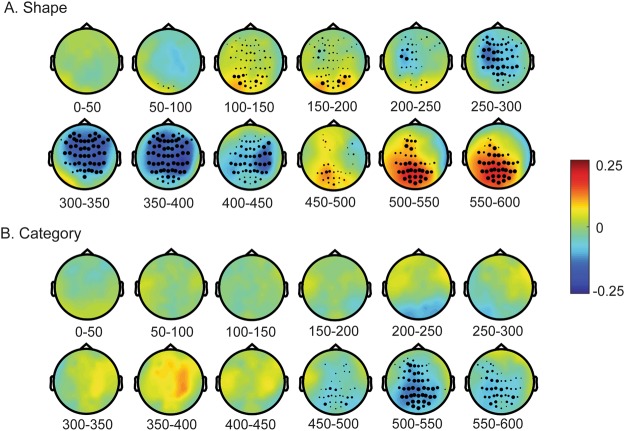
Results of the main cluster-based permutation analysis for Shape (Panel **A**) and the Category (Panel **B**). The colour represents the group mean Spearman rho, averaged within the time interval of 50 ms (time intervals in ms are shown). The black markers over EEG electrode sites indicate that a significant cluster (p < 0.025, see Table 1) included this EEG channel within the given time interval. The larger the marker, the longer the channel remained statistically significant within the given interval.

**Figure 4:**
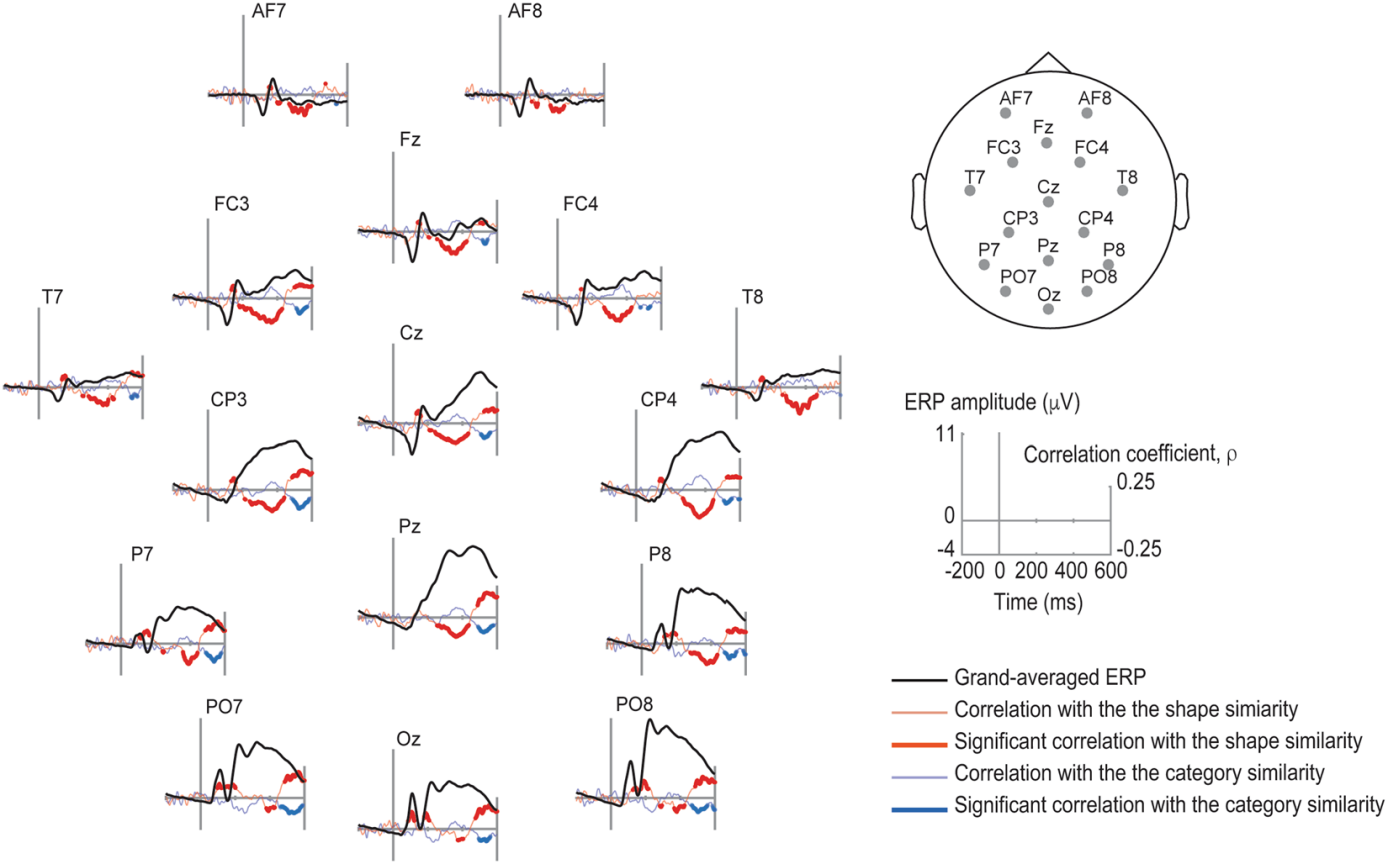
Correlation values plotted against the ERPs. ERPs (black) are averaged over all cue-target combinations over all participants. Correlations with the shape and category similarity in each channel is plotted in red and blue, respectively, with a significant difference from zero, based on the cluster analysis, marked in bold, in red in blue, respectively. A selection of 16 channels (out of original 60) corresponding to the standard 10–20 electrode system is shown.

Table 1 presents the statistics and the temporal extent of the clusters obtained in the permutation analysis. The topoplots on the Fig. 3 illustrate their spatial extent and the waveforms in Fig. 4 illustrate their temporal extend and the correspondence to the event-related response peaks. In the following we describe the obtained clusters that are statistically significant at p < 0.025. Additionally, we obtained several marginally significant clusters. For the sake of comprehensiveness, we report all clusters with p < 0.1 in the Table 1.

**Table 1:** Results of the cluster-based permutation analysis. In the top part of the table (“Main EEG analysis”), results for the cluster with the probability p < 0.1 are shown; significant clusters at the two-tailed cut-off at *alpha* = 0.05 (p < 0.025) are highlighted in bold.

	Probability	Cluster statistic	Standard deviation	Time min. (ms)	Time max. (ms)
**Main analyses**					
Shape	0.0398	−1.6*10^3^	0.003	44	100
	**0.016**	2.2*10^3^	0.002	86	216
	**0.006**	−3.2*10^3^	0.001	174	300*
	**0.0002**	−1.3*10^4^	2*10^−4^	300*	468
	**0.005**	5.9*10^3^	9.9*10^−4^	464	600
Category	0.041	−1.5*10^3^	0.003	184	282
	0.055	2.2*10^3^	0.003	330	420
	**0.006**	−5.0*10^3^	0.001	462	600
**Post-hoc analyses**					
Category (shape-similar pairs excluded)	0.048	−1.5*10^3^	0.003	184	298
	0.338	285.1	0.007	360	392
	0.041	−2.2*10^3^	0.003	452	580
Category (partial correlation, controlling for shape similarity)	0.057	−1.2*10^3^	0.003	186	282
	0.210	591.6	0.006	334	396
	**0.021**	−3.1*10^3^	0.002	474	580

In the bottom part of the table (“Post-hoc analyses”), the updated statistics for the same clusters are shown. *These two clusters reflect the same effect; they are presented separately in the table because the cluster-based analysis was performed separately for the early (up to 300 ms) and late (after 300 ms) time intervals after the picture onset.

The similarity in shape between cues and targets affected the entire dynamics of the visual processing. The event-related signals in the posterior channels starting at 86 ms after the picture onset correlates positively with the shape similarity. As shown by the ERP waveforms in Fig. 4, this cluster in the posterior channels corresponds to the P1 peak or the P1-N1 complex. Next, a large negative cluster spreading over the central regions begins at about 174 ms, followed by a posterior positive cluster after 464 ms.

The results were very different for the category similarity, where the only significant cluster was obtained at a very late time, at 452 ms after the picture onset. Notably, the spatial and temporal extent of this cluster was similar to that of the latest shape similarity cluster. We hypothesised that this effect could be driven by non-independency between the shape and category similarity ratings. Indeed, we found a small but reliable correlation between the similarity and the category ratings: on average, Spearman’s rhos of the participants (M = −0.12 ± 0.11) were significantly smaller than zero (t(19) = −4.95, p < 0.001). We also found that when the eight word-picture pairs most similar in shape were excluded, Spearman’s rhos (M = 0.02 ± 0.11) did not differ significantly from zero (t(19) = 0.8, n.s.). All these pairs (kiwi-potato, banana-zucchini, pear-eggplant, apricot-onion, and the respective reversed pairs) were different in category. Thus, the correlation between the shape and category similarity was driven by these pairs.

To tease apart the effect of shape from that of category, we ran two additional post-hoc analyses. First, we repeated the main EEG analysis, using the partial correlation approach, testing for the correlation between ERP signals and category/shape similarity, while removing the shared variance. Second, we repeated the original correlation analysis while excluding the eight word-picture pairs that drove the correlation between shape and category similarity. The results of the post-hoc analyses are shown in the Table 1. The results of the partial correlation were similar to the normal correlation, however the magnitude of the late category effect has reduced. The second post-hoc analysis yielded a similar picture: the late ERP responses still correlated with the word-picture category similarity, but the effect became smaller and dropped below the significance threshold. This indicates, that the late category effect could be, at least partly, explained by the association between the shape and category in the designed stimuli.

The results of the partial correlation analysis of the shape similarity did not differ from the results of the main analysis and are not shown in the table.

## Discussion

### The effects of shape

Linguistic labels are known to facilitate object recognition, yet the mechanism of this facilitation is not fully understood. A large number of psychophysical studies have suggested that words activate the visual representation of their reference, and particularly its most salient features, such as visual shape^17,19,40^. At the same time, recent visual search experiments have suggested that higher-level semantic aspects of words also affect identifcation of the visual target^30^. In the present work we aimed to tease apart the visual shape and the semantic category effects of words on object recognition, and study the dynamics of these effects at the neural level. We conducted an EEG word-picture matching experiment, using objects from two categories and with four different shapes. We predicted that participants’ reaction times would be explained by both shape and category similarity between the cues and the pictures, and that the effects of shape and category would be dissociable in the timing and topography of EEG. Contrary to our expectations, we found that only the word-picture shape similarity, but not the category similarity robustly predicts the reaction times. The shape similarity also correlated strongly with the ERPs starting in the posterior channels at about 90 ms after picture onset. The timing and topography of this effect (see Figs 3 and 4) are in line with the earlier finding by Boutonnet and Lupyan^22^, who showed, in a similar experiment, that the P1 ERP component was modulated by word-picture congruency. Here we have extended this earlier finding by showing, unambiguously, that this early effect on visual processing can reflect an anticipation of the upcoming visual object shape.

Several recent theoretical and empirical studies have attempted to explain the interaction between language and perception from the predictive coding perspective^10,12,47–50^. Verbal cues are inherently predictive: we tend to talk about objects that we see, and the valid word-object combinations are overtrained by years of language use. Moreover, in the present experiment the words were predictive of the shape of the upcoming object: the probability of seeing a round object following the word “onion” was higher than seeing any other shape, because in 30% of the trials the cue word was followed by a congruent object. According to the predictive coding account, the input in sensory cortices is constantly evaluated in comparison with top-down predictions, or expectations^10,12,47–50^. A mismatch between the prediction and the input results in a “prediction error” response. Anticipated stimuli evoke a smaller prediction error, i.e. a reduced neural response compared to unpredicted stimuli^48,50–54^. Our results, however, are not in line with this prediction: event-related responses over the posterior electrode sites at 86 to 216 ms after picture onset showed a positive correlation with the word-picture shape similarity, most prominently during the P1 (and partly the following N1-P2) ERP components (see Fig. 4). This means that objects with the anticipated shape elicited responses with larger amplitudes. One possible explanation is that in our experiment prediction (i.e. expectations based on the prior probability) was confounded with attention (i.e. task relevance). Indeed, in the present task, participants had to make a decision on the target based on the information provided by the cue, and were thus likely to attend to shape information. The attentional enhancement of the hemodynamic^55–57^ and electrophysiological^58^ responses in the visual cortex is well known. Recent studies have attempted to explain this phenomenon from the predictive coding perspective, by manipulating attention and prediction independently. Indeed, attention reverses expectation suppression in the visual cortex: when stimuli are attended, the neural response is larger in amplitude for predicted compared to unpredicted stimuli^51^. This observation is in line with the response patterns we observed in the present experiment.

Interestingly, we observed an even earlier, marginally significant cluster of correlations between shape similarity and the ERPs, starting at 44 ms after picture onset. The fronto-central location of this cluster allows for the intriguing interpretation that the effect is due to the top-down flow of information from prefrontal attentional control brain areas. The neural correlates of visual attention are well studied in primates. Consider a visual search experiment, where monkeys are trained to search for a target object within an array of distractors. It has been established that neurons in prefrontal cortex respond selectively to the targets, relative to distractors, and selectivity in those areas precedes similar selectivity in the extrastriate and temporal cortex^59,60^. The input from prefrontal cortex thus modulates the target selectivity in the extrastriate areas, enabling visual target detection. Accordingly, the similarity analysis in the present study points toward prefrontal attentional selection that precedes the extrastriate selection: objects with the anticipated shape elicit responses with larger (more negative, see Fig. 4) amplitudes over fronto-central electrodes, resulting in a negative correlation. This suggests a similar mechanism for the language-driven attention control in humans. At the same time, this interpretation is tentative, and the effect should be further investigated.

### The effects of category

As mentioned above, contrary to our expectations we did not find any effect of category of the cue words’ referents on participants’ response times to the target stimuli. However, as we had expected, the effect of category did manifest in target-evoked ERPs. We found a very late category effect, starting at 450 ms after visual stimulus onset. The post-hoc analyses revealed that this effect was still significant, but reduced when the shared variance between the shape and category similarity ratings was controlled for. The effect was, therefore, partly dependent on the shared variance among similarity ratings, reflecting the cue-target pairs that were most similar in their shape (e.g. kiwi - potato; see the EEG results). An attractive explanation for this dependency and the effect’s late latency, is that the greater the word-picture pair’s shape similarity, the more participants had to include category in their decision about whether the cue and picture matched, or not.

Interestingly, weaker effects of category similarity were also found in earlier time windows. Notably, the negative correlation cluster between the ERP data and the category similarity at around 200 ms after the stimulus presentation was still present in the post-hoc tests, and the size of this effect hardly changed with the post-hoc manipulations. The timing and the posterior location of this effect is in line with the congruence effect on the amplitude of the P2 ERP component, observed by Boutonnet and Lupyan^22^. In the present study, this effect, however, was only marginally significant, and thus requires further investigation.

Altogether, our results indicate that the category of a word’s referent might influence processing of subsequent visual stimuli at multiple stages, independent of the referent’s visual shape. We find an interesting parallel with recent neuroimaging studies, attempting to disentangle the contribution of visual and conceptual information to the brain’s object representations. Namely, several recent functional magnetic resonance imaging (fMRI) studies addressed the question if the category selectivity in the ventral temporal cortex can be reduced to the selectivity of visual features, particularly, shape. While some findings support this idea^61,62^, other studies report convincingly irreducible category selectivity effects. For example, similarly to our design, Bracci and Op de Beeck^63^ created a two-factorial stimulus set with images that explicitly dissociate shape from category. Using representational similarity analysis, they identified patterns of fMRI activity associated with the representation of objective visual silhouette, perceived shape and category. Encoding of the perceived shape was closely related to the encoding of category in high-level visual cortex. Nevertheless, the representations of shape and category did not fully overlap; category representation spread more anteriorly in the ventral stream and covered areas of the dorsal stream^63,64^. In another recent study, Proklova *et al*.^65^ compared the patterns of fMRI activity evoked by animate and inanimate objects, in pairs matched for shape, such as a rope coil and a snake. Although the shape feature could well explain the evoked fMRI patterns in the ventral temporal cortex, categorical information, orthogonal to the shape, also contributed to the object representation in more anterior areas.

### Limitations and future directions

Our findings support the hypothesis that words aid processing of relevant visual properties of denoted objects. The present experiment focuses on object shape. Shape is one of the most prominent features for discriminating common objects. Still, it can be less relevant for some objects than for others. Different visual and non-visual features, such as colour or taste, can be discriminative for objects at a more specific, subcategory level, and thus be activated by labels in a corresponding task or context. In fact, the word-picture relationship that we term “categorical similarity” in the present experiment could be a collection of representations in the visual as well as non-visual modalities, such as colour, taste, or sound. Future studies should address the neural dynamics of activation of these features in the language-perception interaction.

Another limitation of our design is that the shape differences between the objects in our study could be greater than the categorical differences. We chose for the close object categories “fruits” and “vegetables” in order to minimise perceptual differences between the categories, e.g. in size and texture. It remains a question if the categorical effects would be more pronounced when the cues and targets are less related, e.g. like the stimuli used by Proklova *et al*.^65^.

## Conclusions

Previous studies have discovered that visual perception can be affected by the top-down guidance of words. Our results advance this line of research by revealing how different kinds of information carried by a word contribute to the different stages of visual processing. We provide evidence that hearing a word can activate representations of its referent’s shape, which interacts with the shape processing of a subsequent visual stimulus. This interaction is detectable from very early on in the occipital electrodes’ event-related EEG signal. We also found that a word’s non-visual categorical information can affect visual processing at later stages: an interaction between the category of the word and the category of the visual stimulus was detectable in the EEG signal much later after visual stimulus onset. These findings provide insight into the interaction between language and perception and into how the meaning of words might be represented in the brain.

## Data Availability

The datasets generated and analysed during the current study are available at data.donders.ru.nl, the online data repository of the Donders Institute.
